# Promoting respectful maternity care: challenges and prospects from the perspectives of midwives at a tertiary health facility in Ghana

**DOI:** 10.1186/s12884-022-04786-w

**Published:** 2022-05-31

**Authors:** Veronica Millicent Dzomeku, Adwoa Bemah Boamah Mensah, Emmanuel Kweku Nakua, Pascal Agbadi, Joshua Okyere, Peter Donkor, Jody R. Lori

**Affiliations:** 1 Department of Nursing, College of Health Sciences, Kwame Nkrumah University of Science and Technology, Kumasi, Ghana; 2 Department of Epidemiology and Biostatistics, School of Public Health, Kwame Nkrumah University of Science and Technology, Kumasi, Ghana; 3 Department of Population and Health, University of Cape Coast, Cape Coast, Ghana; 4 Department of Sociology and Social Policy, Lingnan University, 8 Castle Peak Road, Tuen Mun, Hong Kong; 5 Department of Surgery, School of Medical Sciences, Kwame Nkrumah University of Science and Technology, Kumasi, Ghana; 6 University of Michigan School of Nursing, Ann Arbor, USA

**Keywords:** Respectful maternity care, Midwives, Dignified care, Respectful communication, Ghana

## Abstract

**Background:**

Evidence shows that women in Ghana experience disrespectful care (slapping, pinching, being shouted at, etc.) from midwives during childbirth. Hence, evidence-based research is needed to advance the adoption of respectful maternity care (RMC) by midwives. We therefore sought to explore and document midwives’ perspectives concerning challenges faced and prospects available for promoting RMC in a tertiary health facility.

**Methods:**

We employed an exploratory descriptive qualitative study design. In total, we conducted 12 interviews with midwives educated on RMC. All audio data were transcribed verbatim and exported to NVivo-12 for data management and analyses. We relied on the Consolidated Criteria for Reporting Qualitative Research guideline in reporting this study.

**Results:**

The findings were broadly categorised into three themes: emotional support, dignified care and respectful communication which is consistent with the WHO’s quality of care framework. For each theme, the current actions that were undertaken to promote RMC, the challenges and recommendations to improve RMC promotion were captured. Overall, the current actions that promoted RMC included provision of sacral massages and reassurance, ensuring confidentiality and consented care, and referring clients who cannot pay to the social welfare unit. The challenges to providing RMC were logistical constraints for ensuring privacy, free movement of clients, and alternative birthing positions. Poor attitudes from some midwives, workload and language barrier were other challenges that emerged. The midwives recommended the appointment of more midwives, as well as the provision of logistics to support alternative birthing positions and privacy. Also, they recommended the implementation of continuous training and capacity building.

**Conclusion:**

We conclude that in order for midwives to deliver RMC services that include emotional support, dignified care, and respectful communication, the government and hospital administration must make the required adjustments to resolve existing challenges while improving the current supporting activities.

**Supplementary Information:**

The online version contains supplementary material available at 10.1186/s12884-022-04786-w.

## Background

Globally, maternal mortality is considered an important public health concern. While there has been substantial decline in maternal mortality rate across the globe, several countries including Ghana could not attain the targets of the Millennium Development Goal 5 which aimed at reducing global maternal mortality by 75% by the end of 2015 [1]. Nearly 810 women die every day from pregnancy and child birth related causes globally [2]. Low-and-middle-income countries contribute 94% to the global maternal mortality while sub-Saharan Africa accounts for nearly two-thirds of maternal mortality worldwide [2]. To further advance commitments towards reducing maternal mortality, there was the ratification of the Sustainable Development Goals, of which target 3.1 seeks to reduce maternal mortality rate to less than 70 per 100,000 live births by 2030 [3]. In Ghana, the introduction of the national health insurance scheme, free maternal health care, Safe motherhood, Prevention of mother to child transmission (PMCT) intervention for HIV, folic acid supplementation, coupled with an advocacy for skilled birth attendance and facility childbirth have led to significant improvement in pregnant women’s access to maternity care and reduced maternal mortality [4]. For instance, report from the 2017 Ghana Maternal Health Survey showed that Ghana’s maternal mortality has been improving over the years with a decline from 451 maternal deaths per 100,000 live births in 2007 to 343 maternal deaths per 100,000 live births in 2017 [5]. Notwithstanding, about one-third of pregnant and postpartum women do not access maternity care from health facilities [3]. This poses a significant threat to Ghana’s capacity to achieve the Sustainable Development Goals.

Available evidence suggests that women who do not receive maternity care have reported to have ever experienced or witnessed disrespectful maternity care at health facilities [6–8]. Hence, promoting respectful maternity care has become an important maternal health concern in Ghana. According to Freedman [9], disrespectful and abusive care is the *“interactions or facility conditions that local consensus deem to be humiliating or undignified, and those interactions or conditions that are experienced as or intended to be humiliating or undignified”* (p. 43). Therefore, it stands to reason that respectful maternity care (RMC) denotes the interactions or facility conditions that pregnant and postpartum women deem to be humiliating or undignified in the course of accessing maternity care.

To provide a more standardised definition, the World Health Organisation (WHO) refers to RMC as the organised care that is provided to all women in a manner that does not compromise on their confidentiality, dignity, and privacy while ensuring freedom from harm and mistreatment, as well as enabling women to make informed decisions and assuring them of continuous care from labour through to childbirth [10]. This implies that RMC should include all maternity care services that are devoid of any form of abuse including physical, emotional and psychological abuse [6]. RMC that focuses on individual, cultural, personal, and medical needs of women is vital to improving access to quality maternal healthcare. This is owing to the fact that when women are denied the respect that they deserve, they are less inclined to return to health facilities for future births [6, 7, 11]. The literature is replete with studies that show the existence of significant disparities with respect to who receives RMC [12, 13]. For instance, younger women, unemployed women, and those who give birth in higher level facilities tend to have poorer experiences with respect to RMC [12–14]. Furthermore, most studies that have explored the phenomenon of RMC in Ghana have done so from the perspective of the client.

Respectful maternity care is supposed to be the normal maternity care practice for all healthcare workers. Nevertheless, earlier studies conducted in Ghana have found that disrespectful maternity care often manifested through acts of shouting, pinching, slapping, and physical restraint to a delivery bed, were commonly cited in the labour ward and often exhibited by midwives [6, 7, 13, 14]. Evidence suggests that midwives who show acts of disrespect to women during childbirth do so on the premise of trying to save the mother or baby [6, 15]. For instance, a qualitative study conducted in Ghana [6] and Guinea [15] have both shown that midwives often justified disrespectful care during childbirth when women were disobedient, uncooperative, or to save the life of the baby. Hence, justifying why the study is delimited to midwives. Moreover, with respect to RMC, to the best of our knowledge, there is no empirical evidence that describes the variation in RMC across different cadre of healthcare providers. Evidence available is shows the existence of disrespectful maternity care among midwives [6, 7, 13, 14].

Implementing RMC can enable midwives’ better appreciate care for women in labour and subsequently reduce the incidence disrespectful maternity care [6]. As such, evidence-based research is needed to advance RMC adoption by midwives. Yet, only few studies have explored RMC from the service providers’ perspective, with those studies mainly focusing on midwives’ overall perspectives about the RMC [6]. To the best of the authors’ knowledge and after extensive literature search, no study in Ghana has explicitly focused on exploring the challenges that midwives face in attempting to provide RMC to women, neither has the existing studies explored the prospects and opportunities that exist to promote RMC in health facilities in Ghana. This paucity of literature informed the conceptualisation of our study.

We aimed to explore and document midwives’ perspectives concerning the challenges they face and prospects available for promoting RMC in a tertiary health facility in Ghana. The findings of this study will inform authorities in charge of managing health facilities and other relevant stakeholders about the existing challenges that ought to be addressed in order to improve women’s accessibility to RMC. It will also be important in raising stakeholders’ attention to current opportunities for RMC promotion that might be optimised and strengthened.

## Methodology

### Research team and reflexivity

The research team consisted of midwives and nurses (VMD, ABBM, JRL), social scientists (JO), a surgeon (PD) and bio-statistician (PA, EKN). As such, they had no influence over the study participants and overall data collection dynamics. Moreover, interviewers who could speak both Twi and English language and had vast experience in qualitative research interviewing were recruited to support the data collection process. The research assistants were taken through intensive training for three days in order to deal with any conscious and sub-conscious biases that could have compromised the integrity of the study. The interviewers had no direct influence on the study site, methodological procedures, and findings of the study. As a result of this reflexivity exercise, all of the study authors were able to collaborate more effectively since they were able to grasp each other’s perspectives, which added to the study’s rigour.

### Study design

The study adopted an exploratory descriptive qualitative approach. This design allowed us to explore midwives’ perspectives concerning challenges faced and prospects available for promoting RMC by gathering in-depth information through face-to-face interviews. Qualitative exploratory design served as the most appropriate design for this study since we were concerned about gaining broader and deeper insight about the phenomenon under study. Moreover, we adopted this study design because it allows us the flexibility to respond to varied research questions including questions that border on what, why and how the phenomenon under study happens [16].

### Participant recruitment process and sampling

Purposive sampling technique was used to sample the participants who met the inclusion criteria. The inclusion criteria included the following: (a) participant should have participated in the RMC training, and (b) they should be providing maternity services and willing to participate in the study. In the year preceding the study, midwives at the study site undertook training on RMC. We trained 110 midwives on four separate training period. The training modules taught the trainees how to use effective, alternative birthing positions, focused antenatal care, empathetic and ethical communication with childbearing women, and demonstrating respect and dignity during intrapartum care provision to promote quality intrapartum care free of violence and abuse. Six months after the training, the research team visited the Obstetrics and Gynecology department of the hospital to discuss the study in detail. Two ward-in-charges volunteered as the study’s ‘recruitment links’ Trained research assistants (RAs) visited the hospital to meet with trained midwives through the ward-in-charge as the recruitment link. The study objective was explained to the participants who were eligible, and they were given a copy of the research’s information sheet. We granted prospective participants two-week window to read and review the information sheet in order to guide their decision to either participate or not participate in the study. Midwives with informed decision of participation contacted the RAs via phone for further arrangements on date, time and venue of the interview. Inform consent by writing (*n* = 11) and thumb printing (*n* = 1) were obtained prior to the interviews. It was only after the signed informed consent form had been received that our RAs proceeded to start the interviews.

### Setting

The study was conducted in the maternity block of a tertiary hospital within the Kumasi Metropolis in the Ashanti region of Ghana. This facility is recognised as Ghana’s second largest hospital and the only tertiary hospital in the Ashanti region [17]. It provides healthcare to patients across the country but particularly serves the middle and savannah zones of Ghana. As such, it serves as the primary referral hospital for the Ashanti, Bono, Bono-East, Ahafo, Savannah, Northern and North-East regions as well as some neighbouring countries. The facility has a bed capacity of about 1200 and staff strength of about 3000. It has thirteen (13) clinical directorates (departments) one of which is the Obstetrics and Gynaecology (O &G) directorate, which has four labour wards. In 2018, the hospital recorded an estimated 4792 Spontaneous vaginal deliveries, an estimated 123 maternal deaths, and 61 neonatal deaths [14]. The midwife staff strength at KATH is 381. Table 1 provides statistics on the care provided between 2019 and 2021.

**Table 1:** Statistics on care provided between 2019 and 2021

Activities	2019	2020	2021
Number of births	6814	6247	5909
Number of caesarean births	3033	3174	2982
Number of ANC attendants	–	12,827	14,615
Number of OPD attendants	32,795	22,245	29,446
Average number of births per week	131	118	111
Average number of births per month	568	521	492

### Data collection

ABBM, a qualitative researcher with clinical and academic experience in women’s health and maternal care worked collaboratively with three research assistants (RAs) who had undergone a two-days training about the objectives and procedures for data collection for this study, to conduct the face-to-face interviews with midwives. The interviews were conducted using semi-structured interview guide which was developed based on the WHO’s quality of care framework and an RMC module (RMC-M) developed by the first author in her preliminary studies. For each midwife, we approached them and discussed the objectives and procedures for the study. Additionally, their rights as participants were clearly stated to them as well as any possible discomfort, benefits and compensations. After all these have been explained to the midwife, their consent to voluntarily participate was obtained. All interviews were conducted were conducted as a one-off interview, and at a date, time and place of convenience to participants. The researcher and RAs asked probing questions in order to elicit rich qualitative data for analysis. Data collection began on May 1 through to August 9, 2021. On average, interviews lasted about 70 minutes. All interviews were audio recorded after seeking consent from the participants. In addition to the audio recording, the RAs captured other non-verbal cues and gestures through note taking and observation. By the 10th interview, we had reached saturation as no new analytical information was emerging from the interviews. We conducted additional two interviews to confirm that indeed we had gotten to the point of data saturation. We did not encounter any situation where participants refused to participate in the study. Also, none of the participants dropped out at any point in the study.

### Ethical issues

Ethical approval was obtained from the Committee on Human Research, Publication, and Ethics (CHRPE) at the Kwame Nkrumah University of Science and Technology (KNUST) (reference number: CHRPE/AP/181/18) and the Komfo Anokye Teaching Hospital (KATH) Institutional Review Board (reference number: RD/CR17/289). We anonymised information by giving pseudonyms to the participants in order to protect their identities and prevent third parties from tracing data back to participants. Written informed consent was sought from participants in order for them to voluntarily participate in the study after having read and understood the terms, risks and benefits associated with their participation. Also, the recorded interviews were encrypted to prevent third parties from having access to it. Interview venue (Office at KATH), date and time were determined by the participants. Interview language was Twi (local language). Both the interviewer and researchers could speak and understand Twi on a full professional competence level.

### Data management and analyses

The audio files from the interviews were transcribed verbatim. ABBM proofread the transcribed interviews alongside listening to the audio files as a way of ensuring that, the transcripts reflected exactly what the participants stated. Two independent translators fluent in both the Twi and English languages then translated the twelve anonymised “Twi” transcripts using the process of back-back translation while maintaining confidentiality. Independent thematic coding analysis using QSR NVivo-12 was performed by two data analysts (PA and JO). Translated transcripts were imported into NVivo-12 for data management and analysis. Codes were generated through inductive analysis to create themes and sub-themes. This inductive analysis was done by reading the raw text data and discussing the emerging issues to form themes. Significant recurrent statements or phrases were retrieved as codes from participants’ transcripts to provide data that directly relate to the issue under research. The relevant statements or phrases were then used to develop formulated ‘meanings’ that described and illuminated the obstacles and opportunities for promoting RMC. Following that, themes were created based on various statements with comparable meanings. This process was repeated for all the 12 transcripts. Insights from the transcripts were broadly presented in line with the main questions in the semi-structured guide. To completely develop the ideas, the original themes were followed in subsequent interviews and validated using field notes. The initial analysis was performed by PA and JO, and later validated by the first, second, and third authors and through member checking with five participants. These participants reviewed the printed transcript so as to confirm the accuracy in the presentation of their views. Member checking allowed us to confirm the findings from our analysis. However, none of the issues changed after member checking.

### Rigour and trustworthiness

Recognising the worth of rigour and trustworthiness in qualitative research, we ensured that our study and its methods adhered strictly to the principles of credibility, confirmability, transferability, and authenticity. Transferability was ensured by giving detailed description of the study objectives, research design, data collection procedures, study contexts and data analysis procedures. Confirmability was ensured by allowing five of the participants to review the printed transcript so as to confirm the accuracy in the presentation of their views. To authenticate the results, completed interviews were first reviewed by the interviewers. After that level of review, VMD, who is project lead and an experienced qualitative researcher together with ABBM and EKN validated the results. To ensure credibility, we adhered strictly to the study protocol and ensured that audio data were transcribed verbatim.

## Results

### Demographic characteristics of participants

Table 2 shows the demographic characteristics of the sample. The ages of the participants ranged between 26 and 50 years, with the median age being 35 years. With the exception of participant 1 (P1), all the participants had attained a degree. Of the twelve participants interviewed, seven of them, representing 58.3% were married. Fifty percent of the participants had no child. Also, 67% of the participants sampled had between 1 and 5 years’ work experience as midwives.

**Table 2:** Demographic characteristics of participants

Participants ID	Age	Educational attainment	Marital status	Parity	Years of experience
P1	34	Tertiary (Diploma)	Single	0	5
P2	37	Tertiary	Married	3	12
P3	35	Tertiary	Single	0	11
P4	34	Tertiary	Married	3	5
P5	38	Tertiary	Married	0	3
P6	38	Tertiary	Single	0	5
P7	32	Tertiary	Married	0	2
P8	28	Tertiary	Single	0	4
P9	51	Tertiary	Married	3	11
P10	35	Tertiary	Married	3	11
P11	26	Tertiary	Married	1	1
P12	30	Tertiary	Single	1	4

Source: Fieldwork, 2021

### Main findings

Midwives were expected to provide care to childbearing mothers in a supportive, respectful, and dignified manner. This type of care—respectful maternity care—is centred around three main themes in this study: emotional support, respectful communication, and respectful and dignified care. The provision of this care, like any other caregiving, requires both the expertise and the enabling environment for its sustainability. This study gave the opportunity to a handful of midwives to express their knowledge about specific respectful maternity care activities they are expected to implement and to equally detail any person-level or structural-level factors that may derail the performance of RMC activities. The findings are reported under the following categories: current actions undertaken, challenges and recommendations to improve RMC provision (see Table 3).

**Table 3:** Summary of main findings

Themes	Sub-themes		
	Actions	Challenges	Recommendations
Emotional support	• Provision of sacral massages and reassurance • Encouraging male involvement	• Inadequate staff • Non-cooperation from women • Partner’s lack of courage • Inadequate material resource and limited space	• Increasing staff strength • Logistical support for a timely response
Dignified care	• Ensuring confidentiality • Provide information and seek consent • Referring mothers who cannot pay bills to social welfare	• Logistical constraint on alternative birthing positions • Facility environment limits privacy and movements • Clients’ condition limits movement	• Logistical support for alternative birthing • Capacity building and training • Logistical support for privacy and free movement • Motivating midwives
Respectful communication	• Ask relatives to excuse patient-provider conversations • Introducing ourselves as caregivers	• Poor attitudes of midwives • Language barrier	• Peer monitoring • Demonstrating good attitudes

### Providing emotional support

#### Actions implemented to provide emotional support

##### Sacral massages and reassurance

Emotional support can be provided in the following way: performing sacral massage, allowing birth companions, and responding timely to the needs of the childbearing women. Evidently, childbearing women are often plagued with many thoughts, including those of fears and anxiety. Thus, midwives are expected to reassure them of the childbirth process, by performing sacral massage on the woman in a comforting manner and counselling her to alleviate fears. From the responses on this theme, it was evident that the midwives involved family members of the childbearing women in the process of providing emotional support. For instance, one of the participants explain how she performs the sacral massages and the reassurances, highlighting family members involved in the process:

*“Usually, women in labour are tensed and anxious. As such, it is critical to de-stress them and make them feel comfortable. So, what we do is that, we give them sacral massages (locally called apemfo amamia). In addition to that, we give them words of encourage so that they (woman) will feel reassured.”* **(P10, 35 years).**

The involvement of family members was evident in the performance of sacral massage and reassurance activities. The participant said that, *“at times we seek the support from the husbands to give them [their wives] the sacral massage, encourage and reassure them”* **(P3, 35 years).**

One of the participants explained that first-time mothers are often afraid and anxious about the childbirth process because of what they heard from others about the birth pangs. In such situations, the midwife mentioned that describing the stages in the birthing process can help the childbearing women be at ease. She explained how she goes about this in the following way:

*“Some of the patients are emotional. They are often fearful and anxious. Possibly, it is because they have not given birth before but may have heard that the process is painful. For some other clients, the way people may have described the process to them would have put a frightening aura around the birth process for the. In such instances, we try to calm the woman by encouraging and assuring her that she is in safe hand, and that everything is going to be alright. We keep her informed by describing the stages of labour to her. This helps us to calm the person and gain their cooperation because they are now aware of all the stages of birth and all other expectations.”* **(P4, 34 years).**

##### Encouraging male involvement

Allowing birth companions enable the childbearing women to be comfortable given that they might find it easy to share their worries and concerns with these companions. Giving opportunity to childbearing women to be with birth companions of their choosing is another way to create an enabling environment for emotional support. The participants usually allow partners of the childbearing women to be with them during the birthing process. The midwives explained that they allow the men to be present so they can learn to provide compassionate care to their wives whiles they are in the hospital and when they return home after a safe childbirth.

*“Over here, we try as much as possible to involve the males throughout the process. As such, we normally encourage them to come in and support the women. You know; women can be more cooperative when it is their husband who is giving them sacral massage or when it is their partner who is encouraging them to push the baby out. So, we encourage room-in and involve the men the birthing process”* **(P7, 32 years).**

##### Challenges

There are challenges to providing optimal emotional support to childbearing women. The challenges are related to inadequate staff, childbearing women attitudes, difficulty dealing with family members of the childbearing women, and hospital infrastructure deficits.

##### Inadequate staff

The midwives mentioned that a limited number of midwives resulting in unbearable workloads prevent them from providing emotional support in the best way they should. Regarding inadequate staffing and workload effect on the provision of emotional support, these midwives have the following to say:

“*… it got to do with the inadequate staff. If we’re not busy we can attend to the patient promptly but if we are two (2) on duty and we are all busy we can’t attend to the third person. We attend to the emergency cases and later to those that can hold on with their condition but if there were to be enough staff, we can attend to all”* **(P1, 34 years).**

*“… with respect to emotional support, it is quite challenging because of our staff strength. There could be more labour cases which makes it difficult to promptly attend to a patient’s emotional support one after the other but we reassure them”* **(P3, 35 years).**

*“… sometimes, here [referring to the hospital], we do seventeen (17) deliveries at night so in case there’s workload and you call we can’t attend to you; [this is] not intentional though, but the midwives [are] not enough”* **(P11, 26 years).**

##### Non-cooperation from women

The next sets of challenges disabling midwives in their quest to provide emotional support to childbearing women include non-cooperation from women and partners’ lack of courage. Regarding the challenge of non-compliance, the midwives express their concerns as follows:

*“Some patients don’t comply with instructions. How do we apply the massage? We do the sacral massage but some mothers don’t cooperate so we can’t do it”* **(P11, 26 years).**

*“Let’s say there are some patients, you approach them in this way: ‘Ma’am, the pain you are going through …*. *especially after labour has started …. that is what propels the baby down the birth channel to the outside, but no matter what you say to such a patient, she will ignore you. No matter what you say, she will ignore you. It is rather what your advice against that she will actually do …. And it seems like whatever you’ve been saying to her does not really register in her mind but rather passes over her ears. So, it can cause you to be reluctant: you can actually make up your mind not say anything further”* **(P5, 38 years).**

*“What I can say is, it is also their relatives. I don’t know, because this facility is a referral centre when they are being referred and then they come here, even when it is not labour, I mean it is a condition they came in with, the relatives don’t give us that space. You tell them to, maybe the patient is in pain, they are around, you tell them (relatives) to go, they (patients) are in your hands now, you are taking care of them, together with the doctors. Every point in time, you see a relative trooping in, especially when they see that the patient that they brought, the relative that they brought, the person is in pain, they (relatives) want to be involved in whatever is going on. I am not saying that they shouldn’t be involved, but I think so far as you’ve brought the patient into the facility, you’ve left her in the care of the doctor and the midwife or the nurse. So, whatever is going on, they should just allow us to do our job. Yeah, they should help us do it as they should just give us that space, that room to perform our jobs and after that, we will communicate whatever is going on to them”* **(P8, 28 years).**

##### Partner’s lack of courage

Some of the participants mentioned that partners of childbearing women lack courage to provide emotional support as they often felt uncomfortable witnessing the pain their wives go through during the childbirth process.

*“Many men don’t like it that way they don’t want to see their wife going through that pain. I remember I invited a husband and he even collapsed before seeing the baby’s head (laughs). Some will refuse if you offer the opportunity because he can’t watch. Few ones that are eager we have a way of letting them in. I gave some husbands the opportunity”* **(P2, 37 years).**

##### Inadequate material resources and limited space

The other last set of challenges mentioned by the midwives are related to inadequate material resources and limited space. They mentioned that the hospital has no waiting area for relatives of childbearing women and there were only two delivery beds. These limitations prevent them from providing emotional support.

*“So far, the hospital has no waiting room for relatives so if they come, they’ve to wait outside”* **(P1, 34 years).**

*“We have two (2) delivery beds in one room and two (2) women delivering at the same time with no curtains. Now here we have curtains. But the entrance is the problem where the husband will pass because the labour ward is connected to the theatre so entry is a problem*” **(P2, 37 years).**

*“So, if two (2) or three (3) ladies become ‘full’ simultaneously, we will do the others on beds, and the others right here (indicating). Therefore, for the ones that we have to perform our duties on a bed, the husband cannot practically be there, because, right beside the wife, there will be another patient lying there. If you do that, you will be invading someone else’s privacy”* **(P5, 38 years).**

##### Midwives’ recommendation for enhancing emotional support

The midwives suggested that the implementation of the following solutions will enable them to provide the necessary emotional support to childbearing women: increasing staff strength and logistical support for a timely response.

*“More staff needs to be employed. We cannot have one (1) the patient is to one (1) midwife but at least there should be extra so that activities can be shared among us”* **(P1, 34 years).**

*We need to increase staff and some staff needs to change their attitude. We are not the same. Some will work hard others will not so mostly the patient expect the hard workers to treat them but you may be tired. We have to advise ourselves not all of us but at times we are the problem* **(P12, 30 years).**

*We need more equipment with foetal heart monitor we have two (2) so in case there are three (3) labour cases it means you’ve to use the manual one for the other patient which requires your presence but for the electric one you can listen to the feedback while doing other things. It will help us if we had enough and at times, we don’t get the number of consumables we request for. Meanwhile, the one been provided is not enough for the unit work* **(P3, 35 years).**

### Provision of dignified care: awareness, challenges, and recommendations

#### Actions implemented to provide dignified care

##### Ensuring confidentiality

Participants commented on how ensuring confidentiality was an integral way of promoting and providing dignified care to women in labour. The participants acknowledged that they asked sensitive questions only when they are alone with the client in their cubicle. This eased women and ensured that there is dignity and respect throughout the care process. In situations where it was almost impossible to ensure confidentiality, midwives reduced their tone in order for third parties not to reduce the possibility of accidentally disclosing information to third parties. This is reflected in the quotation below:

*Oh, in such situations, I will be able to admit you and put you in a bed and then when I am taking the FH, your vitals and such inside, I can ask you that. It doesn’t exactly have to be at the time of admission that I have to get all the required information, for when I am taking the vitals, the relative is not with us. Uh huh, so I am alone with you in the cubicle, ‘OK, Sister, please, for the medications, have you started treatment? Have you done this or that?’ and when we get to such a stage, the client is capable of telling you everything, knowing it is just between the two of you* **(P4, 34 years).**

*If you can’t provide privacy, you have to talk in a lower tone because if it is abortion and you alarm it for relatives to hear they can divorce her. All you need is to talk under tone to prevent others from hearing* **(P9, 51 years).**

##### Provide information and seek consent

In providing dignified care to women in labour, there should be absolute non-compulsion. The participants alluded to this as they revealed that in providing services and care to clients, they always provided them with sufficient information about that particular care in order for them to make an informed choice and consent to their participation. Below are direct quotations from the participants:

*If it is MagSulf (magnezium sulphate) – MagSulf is given every four (4) hours – you tell the patient that …. they, the patients refer to MagSulf as ‘the needle that extremely burns’, so ‘Madam, I am about to give you the injection that is extremely painful or burns. You seek for their consent before you do that: everything you do for a patient; I think you have to explain and seek consent* **(P8, 28 years).**

*Like …. Mmmmm … … I see it that anything you have to do, you have to make the client aware, you have to tell her and explain what you are about to do and why you are to do it, if she will give you the permission* **(P10, 35 years).**

##### Refer mothers who cannot pay bills to social welfare

The midwives indicated that there are times that women who come to deliver are unable to pay their bills. This makes it difficult for them to enjoy satisfaction of care. Hence, to ensure their dignity amidst their financial constraints, midwives refer the client to social welfare for further assistance to offset their bills. The quotes below are synopses of midwives’ account of referring mothers who cannot pay their bills to social welfare:

*If a client cannot pay her bills, we turn her over to the social welfare department. Yeah. So, we no longer detain them as we use to … ….* **(P5, 38 years).**

*We come in when the patient tells us that maybe ‘I was charged a thousand cedis (1000GHC), I have five hundred (500GHC). So our new approach as midwives is to involve the social welfare department … … ’. So, we refer them to social welfare. Then they take it over from there. But before the RMC training, we use to detain them …… yes …. of course, because that is the hospital protocol* **(P8, 28 years).**

Nevertheless, there some participants opined that there were times that they could not allow the women to leave the facility until they had an assurance of payment.

*Hmmm right now if a patient delivers and she has no money to pay it is your shift that you’ve discharged her and the link system your name and everything is on it so if there’s any follow up, they’ll know you’re the one that didn’t collect money and the hospital will ask you to pay so a midwife won’t put herself in such situation so you pay before you leave* **(P2, 37 years).**

#### Challenges

In the quest to deliver dignified care to women in labour, midwives encounter some challenges ranging from logistical constraints on alternative birthing positions, facility environment limiting privacy and movements, and unavailability of drapes.

##### Logistical constraint on alternative birthing positions

From the accounts of the midwives, the hospital lacks the necessary logistics to support alternative birthing positions. This makes it difficult or impossible to explore and practice other birthing positions besides lithotomy. This is what some of them had to say:

*We don’t have the necessary equipment and the way clients may behave, if we decide to improvise on the squatting, it is not going to go well. It will not work well at all* **(P7, 32 years).**

*There is position like the squatting position but here we don’t have the resources to support the squat birthing. We are used to the lithotomy and all the delivery beds are in that form. We would have adapted to patient’s preferred position such as squatting if we had the needed equipment* **(P2, 37 years).**

*For water birth, hmmmm … … we are aware of such position. However, we don’t have it [equipment] here. Nothing even shows that we are actually preparing ourselves to do water birthing! Should a patient request for this, then, hmmm …… we cannot meet this need. Let us see what the future holds for all other birthing positions.* **(P1, 34 years).**

##### Facility environment limits privacy and movements

Ensuring privacy is extremely important for childbearing women. However, the midwives expressed their concern about the facility’s environment to ensure privacy and movements of women in labour. According to the midwives, they lacked the necessary logistics and environmental context to promote privacy of childbearing women. They asserted that the available space at the hospital was too small, and that women were housed in open labour wards.

*… … ahaaa, so they are all bunched together inside and if you are working on a patient and you even use the screens, there are holes within the screens so someone can see through these (holes) and take a peep at naked patients. This too doesn’t help us* **(P5, 38 years).**

*the environment is not spacious enough to accommodate fee movement. There is always overcrowding due to the patient turn out. Hence, so we restrict walking around* **(P9, 51 years).**

Closely tied to the issue of limited privacy is the unavailability of drapes. Some of the midwives mentioned that there were no drapes at the hospital to cover clients during childbirth. As such, they had to improvise by using the clients’ own clothes. This epitomised in the following quotes:

*Ideally, the hospital has to provide drapes but we don’t have so we cover clients with their own sheet. Assuming the client comes in an emergency with no clothes, then ensuring privacy becomes a challenge* **(P3, 35 years).**

*As for the drapes, we don’t have any in this ward. So, the alternative we have here is the screen. So, we screen the patient and we do our thing* **(P5, 38 years).**

*The drape itself isn’t available (smiles) and let say the ward has only three (3) and it has been used. With my previous ward, the screens were scarce and even if it is available, it is faulty. Thus, providing privacy becomes a challenge* **(P1, 34 years).**

##### Clients’ condition limits movement

Being able to move freely as one wishes to is fundamental to patients’ feeling of autonomy. Therefore, any restriction in the client’s movement could be viewed as limiting their dignity and autonomy. It emerged from our analysis that the condition of women in labour tends to limit their capacity to move about at the hospital. Specifically, women who had ruptured their membranes were restricted from moving for fear of cord prolapse and other complication. This was considered as a challenge to providing dignified care to childbearing women. The following quotes reflect the views of the midwives:

*Yes, when the fluid comes out so you can’t allow her to move about with the fear that as the fluid leaks it could rupture for the cord to slip to cause cord prolapse that’s why we restrict them but if everything is fine without rupture, they’re allowed to move around because the force of gravity is even necessary so we encourage them to walk* **(P3, 35 years).**

*Well, when it comes to walking, you are allowed to walk but it depends on each stage and what you expect from the client. Let’s say the client is at eight cm (8 cm) … 8–9, at that stage, this is difficult. It is a transitional stage and she is traumatized and such, there is also the possibility of prolapse, and other complications too* **(P4, 34 years).**

#### Midwives’ recommendation for improving dignified care

We sought to understand midwives’ perspectives about the essential recommendations necessary for improving dignified care. They recommended logistical support for alternative birthing, capacity building and trainings, logistical support for privacy, and motivation of midwives.

##### Logistical support for alternative birthing

To ensure the implementation of alternative birthing positions in order to improve dignified care for childbearing women, midwives recommended that the hospital must equip them with the necessary supportive logistics. The voices of the participants are captured in the following quotes:

*The hospital has to acquire all the necessary equipment that will enable us to practice alternative birthing like the water birth* **(P1, 34 years).**

*We need the beds and the instruments they will use for those positions. As long as we have those, we will allow them to use them* **(P10, 35 years).**

*Oh OK, we have to get the different equipment performing those positions, like the birthing chair you talked about when we came (for the workshop)* **(P4, 34 years).**

##### Capacity building and trainings

The midwives also recommended that in order to improve dignity of care, there is the need for the hospital management to organise workshops and training for them on alternative birthing positions.

*Then, there should be a training on that [alternative birthing positions] for midwives. Though we’ve been taught about the new ones in school but we never practiced it so if the hospital is to provide it has to organise workshop for us* **(P1, 34 years).**

*when I had not yet come (to the workshop) to learn about respectful care, some of my attitudes and behaviours were not really optimal but once I came and got educated on these, things have changed. And a lot of things have changed. Therefore, we should go to the district, and other sectors and educate them the more, and even if it is possible, let us take the education to the schools so that by the time the person is coming (the healthcare worker is being sent to the ward), she would have picked it (the desirable concepts of respectful maternal care) already. And we need to continually re-educate, so our minds would be drawn to certain things all the time* **(P10, 35 years).**

##### Logistical support for privacy and free movement

Given that midwives experienced challenges with ensuring the privacy and free movement of women in labour, they requested that the hospital management provide them with supportive logistics such as curtain, individual cubicles or rooms.

*Yeah, when the screens are not enough, we can use the curtains. All you have to do is draw the curtains. So, if we were provided with more curtains or if they gave us more screens, we could ensure privacy. But albeit the problems, we do ensure privacy all the same* ***(P6, 38 years).***

*Maybe in our ward, we should separate the labour cases from our ward to a different place, or let’s say for Cubicle One (1), let’s make it into a cubicle for only labour cases. So, we can use curtains to separate the place into individual rooms for each client that comes in. So, if you are in labour, your relative can stand beside you during the process* **(P5, 38 years).**

##### Motivating midwives

The midwives also stated that in order for them to provide dignified care, there is the need for the hospital management to provide motivation to midwives.

*Mm we have talked about all but I will add up that the hospital should add a bit of motivation to staffs although they are being paid for all they do but a bit of motivation will do to encourage us because at times you will realize they don’t appreciate what we are doing* **(P1, 34 years).**

### Provision of respectful communication: awareness, challenges, and proposed solution

#### Actions that promote respectful communication

##### Ask relatives to excuse patient-provider conversations

In describing actions that supported respectful communication, the midwives mentioned that they asked the woman’s relatives to excuse them whenever they had to discuss or solicit confidential information form women in labour.

*When referrals come at the same time. Even if is second stage we attend to them first before we take other history, at times we ask other patient to excuse us if only the energy is there to wake and excuse us* **(P2, 37 years).**

*So, you just have to excuse the relatives … allow the relatives to excuse you so that the woman can be, the person can be truthful enough to tell you whatever (there is). Because, some people wouldn’t feel comfortable if their husbands or their spouses are around, or their family members, their relatives are around to tell you whatever, especially when they have their top-sending* **(P8, 28 years).**

*But if she sees a relative around … I walked up and told the relatives to excuse us for a while so we can get closer to the client and take better care of her, but they got offended even though I did not say it in a bad way* **(P4, 34 years).**

##### Introducing ourselves as caregivers

From the midwives’ perspectives, introducing themselves as caregivers to women who are there to give birth was a way of promoting respectful communication. The following quotes exemplify this finding:

*For communication we’re very good at it. After shift handing-over, we introduce ourselves to them so if they’ve problems they complain to us if we need to call a doctor we do if we can handle it, we do the needful* **(P3, 35 years).**

*Yeah. So, when we introduce ourselves to the clients, even if not by name, then when you go to perform a procedure for the client, she already knows you are part of the staffs and so she already has confidence in you. Then she goes like ‘Madam, what is your name?’ and you tell her your name and maybe she also says ‘I am also called this’. Ahaaa … .and then you proceed to conduct the care you have to give to her* **(P4, 34 years).**

#### Challenges

##### Poor attitudes of midwives

Some midwives revealed that the attitudes of their colleagues as a challenge to promoting respectful communication in the discharge of their service or care to women in labour. They mentioned that some midwives called clients by the name of their condition.

*At times (laughs) let me say is their character or some encounter she had before come to work so it annoys her seeing you the patient. The moment you ask question she will scold you not really good but not all of us are like that so we have to advise ourselves* **(P12, 30 years).**

*Some midwives call patients by condition they have. Professionally it is not done. A patient has a real name and must be called by that not her condition. We have to put a stop to that it is a condition and won’t stay forever* **(P9, 51 years).**

##### Language barrier

Also, the midwives indicated that language was a barrier in promoting respectful communication in delivering RMC.

*In some cases, it is the language; there are situations in which the language being spoken by the client is not understood by me. She speaks a different language: she doesn’t speak Twi and neither does she speak English. In a scenario like that, it has to fall on the family member present who speaks the language (of the health worker). So, you speak to the relative, and the relative then translates for the client* **(P10, 35 years).**

*Erm...it depends on the level of communication of the relatives maybe language barrier you’ve to explain things to their understanding but at times you’ve to seek the patients consent rather so that she can relay the information to relatives to her satisfaction* **(P2, 37 years).**

#### Midwives’ recommendation for improving respectful communication

##### Peer monitoring

From the study, the midwives mentioned that there is a possibility for their colleagues to sometimes act unprofessionally and compromise respectful communication. Therefore, to remediate this, it is essential for them to be each other’s keeper through peer monitoring.

*Hmm … for now I think we have to be each other’s keeper. There may be somethings that you will do and it will feel odd. But if we check on each other and become each other’s keeper, then we will be able to help ourselves at the ward. We can talk to ourselves, and also involve the senior midwives so that each student nurses and rotational nurses will be practicing professionalism in communicating to patients when they come here to give birth. All these are taught in school and doesn’t need any workshop or training. If you treat a patient right, they deem it much offer you’ve done them.* **(P2, 37 years).**

*No but with respectful maternal care we’ve to be each other’s keeper so patients should take it easy with us in order for us to deliver care to them* **(P3, 35 years).**

##### Demonstrating good attitudes

The midwives also mentioned that in order to ensure respectful communication in course of delivering RMC, it essential for all midwives to demonstrate attitudes that are supportive and not disrespectful. This is reflected in the quote below:

*We don’t need anything special but it is up to us to have good attitude and know how to deal with patients. We have to educate them on when to ask questions it may seem emergency to them but they’ve to bear with us. Most of the problem is our attitude so no matter what we’ve to portray good attitude towards our patient* **(P3, 35 years).**

## Discussion

This study sought to explore and document midwives’ perspectives concerning challenges faced and prospects available for promoting RMC in a tertiary health facility in Ghana. The findings were broadly categorised into three themes: emotional support, dignified care and respectful communication. For each of the themes, the current actions taken to promote it, its challenges and recommendations were captured. Overall, the current actions that promoted RMC included the provision of sacral massages and reassurance, ensuring confidentiality and consented care, and referring clients who cannot pay to the social welfare unit. The challenges to providing RMC were logistical constraints for ensuring privacy, free movement of clients, and alternative birthing positions. Poor attitudes from some midwives, workload and language barrier were other challenges that emerged. The midwives recommended the appointment of more midwives, and logistics for provision of alternative birthing positions and privacy. Also, they requested continuous training and capacity building.

Our study revealed that performing sacral massages and responding timely to the needs of the childbearing women are ways midwives provide emotional support to clients. This result is corroborated by previous studies [18, 19] that have found the use of sacral massages as a means of providing emotional support to women in labour. A plausible explanation for this observation could be that, during labour, women experience excruciating pains that tends to push them to a state of anxiety. However, the application of sacral massages significantly reduces labour pain and feelings of anxiety while simultaneously enhancing women’s satisfaction with labour [18]. From the study, midwives occasionally allowed birth companions into the labour rooms as a way of ensuring the emotional stability of clients. This finding is synonymous to that of a related study [20] that revealed that the presence of birth companions provided emotional support to labouring women.

Consistent with the findings of previous studies [21, 22], we observed that midwives considered asking sensitive questions only when they are alone with the client in their cubicle, providing information and seeking consent as actions that promoted dignified care. Similar result was reflected in our findings that, asking relatives to excuse women and service providers during professional conversations was an action implemented to promote respectful communication. A plausible explanation for this observation could be that, non-consented and non-confidential care are basic elements of disrespectful care and undermine the dignity of women in labour [23]. Therefore, ensuring confidentiality and consented care becomes paramount in promoting dignified care. Nevertheless, providing information and explanation of procedures to gain consent was not always done by midwives due to workload of midwives. This concurs with the findings of a qualitative study conducted in Ethiopia [24].

Although the midwives alluded to the existence of certain supportive actions that promote emotional support, dignified care and respectful communication, they expressed concerns about some existing challenges. Notable among the challenges identified was inadequate logistical support for promoting alternative birthing positions and ensuring privacy. This finding is synonymous to an earlier study conducted in Ghana [17] that found logistical constraints for alternative birthing positions and limited resources to ensure privacy for childbearing women as one of the major challenges confronting midwives in their quest to promote RMC. Thus, emphasising the need for the hospital management to ensure sufficient resources such as drapes and screens are provided towards ensuring privacy of women who come there for childbirth. Also, the midwives recommended that in addition to the provision of equipment needed to support alternative birthing positions, it is imperative for the hospital management to provide capacity building and continuous training for midwives about alternative birthing positions. This finding is supported by a related study conducted in Tanzania [25].

Another challenge that emerged from our analysis was the low staff strength at the hospital. Midwives in this study asserted that the low staff strength made it difficult for them to sufficiently provide emotional support to women in labour. The result is analogous to that of an earlier study in Kenya [26] wherein the authors found staff shortages as a principal challenge to providing RMC. As such, the participants recommended that the hospital management need to increase the number of midwives in order for them to provide sufficient emotional support to women in labour. Our findings also showed that there were times that clients had to be detained because they could not afford their bills; this corroborates with previous studies in Nigeria [27], sub-Saharan Africa [28] and among low-and-middle income countries [29]. Probably, when the midwife allows the woman to leave the facility without settling their bills, then they (the midwife) will be responsible for paying of the woman’s bills. This may explain why women who cannot afford their bills are sometimes detained.

The present study showed that the clients’ condition limited their capacity to move freely at the hospital, and this was considered a challenge to the provision of dignified care in the continuum of RMC. Women who had ruptured their membranes were restricted from moving for fear of cord prolapse and other complication. Existing literature [27, 28] has documented similar findings where the condition of women in labour has restricted them to their bed space for fear of cord prolapse and other complication such as foetal distress. We postulate that such immobility can be a trigger for disrespectful abuse. This is because, midwives in their quest to prevent cord prolapse and other complications among women at that stage of labour, may be tempted to shout, physically restrain women or even use abusive tone and words to dissuade women from moving around [27].

We also found that some midwives demonstrated unprofessionalism and unsupportive attitudes that hampered the provision of respectful communication and dignified care. Some midwives called women by their conditions and this is a clear demonstration of disrespectful maternity care. This substantiates findings from previous studies conducted in Nigeria [29, 30] and Ghana [31]. The observed disrespectful maternity care could be explained from the perspective that, midwives may have premeditated or preconceived perceptions about how women in labour should behave. As such, women who are considered uncooperative with procedure become a source of stress for midwives; that frustration can lead them to maltreat or be disrespectful towards childbearing women [27, 32].

Policy wise, our study has some implications. The findings from the study indicate that there are micro, meso and macro level factors that either facilitate or challenge midwives’ capacity to promote RMC. This calls for Ghana to consider RMC intervention implementation modules that takes into account the various levels of challenges and opportunities to be leveraged. Perhaps, Ghana can take cue from Kenya’s Heshima Project [33], or Tanzania’s Staha Intervention [34]. Both interventions acknowledge that RMC is beyond the interpersonal and clinical components of care. It requires structural/institutional commitments, policy backing, and human resource recalibration. Hence, continuous professional development would be to be implemented in order to reduce the occurrence of disrespect [35]. Our findings also emphasise the need to champion patient-centred care and a rights-based approaches in promoting RMC.

### Strengths and limitation

The rich description of the study context is one of the strengths of the study, as it allows it to be transferrable. Also, the contributions of two experienced nurse-midwives, VMD and ABBM, in the study ensured the validity of the interviews conducted and the results generated. Thus, the credibility of our findings would be difficult to override. Nonetheless, there are some limitations that are worth mentioning. First, the sample frame used was limited to midwives who had participated in prior RMC trainings. Also, the use of only a qualitative method to explore the phenomenon does not provide the capacity of the study to be generalised to all midwifery contexts. Given that the interviews were conducted at the hospital setting, there is the possibility of social desirability bias because they may want to tread cautiously with their responses in order not to implicate themselves or their colleagues. We were able to minimise the likelihood of social desirability bias by reaffirming the confidentiality and anonymity clauses in the execution of the study. Irrespective of the limitations, the study was able to elucidate the current actions, challenges and recommendations for promoting RMC.

## Conclusion

The goal of this study was to explore and document midwives’ perspectives concerning challenges faced and prospects available for promoting RMC in a tertiary health facility in Ghana. We conclude that in order for midwives to be able to provide undiluted RMC services through the provision of emotional support, dignified care and respectful communication, there should be continuous training and capacity building, motivation of midwives, as well as provision of logistics and equipment necessary for supporting alternative birthing positions, privacy and free movements of women in labour. We therefore recommend that, policies and programmes aimed at enhancing RMC delivery must address the various shortcomings while strengthening existing facilitating practices such as the provision of sacral massages and reassurance, ensuring confidentiality and consented care.

## Supplementary Information

**Additional file 1.**

**Additional file 2.**

## Data Availability

All transcripts of the interview that was used for the analyses in this study are available from the corresponding author on reasonable request.

## Abbreviations

DMC: Disrespectful Maternity Care
O&G: Obstetrics and Gynaecology
RAs: Research Assistants
RMC-M: Respectful Maternity Care-Module
RMC: Respectful Maternity Care
SDG: Sustainable Development Goals
SSA: Sub-Saharan African
