# Supplementary material for: Promoting respectful maternity care: challenges and prospects from the perspectives of midwives at a tertiary health facility in Ghana
Source: BMC Pregnancy Childbirth. 2022 May 31;22:451. doi: 10.1186/s12884-022-04786-w (PMC9153163; doi:10.1186/s12884-022-04786-w)
Supplement: Supplementary file 1 — Additional file 1. [file 12884_2022_4786_MOESM1_ESM.pdf]

# CODEBOOK

| EMERGING THEMES FOR RMC PROJECT – MANUSCRIPT 1 | | | |
|---|---|---|---|
| **Themes** | **Files** | **Ref.** | **Quotes** |
| *Actions that promote emotional support* | | | |
| Counselling and education | 2 | 2 | We explain to them that this is how the processes proceed. So, let's say after every VE (Vaginal Examination) that is conducted, you tell the patient, this is how much your cervix has been opened up to, and this is how far we've got to go, and the time left in front of you, this is how far the pain may amount to'. So, in all, she must endure the pain a little while, and move things gradually, and God will deliver her eventually. Then, you will be talking to her…..because you know most at times for the primips, for them, they've never experienced this kind of pain before, so they act differently from those who have already given birth before, so it is the continuous counselling and education that you have to keep giving to the patients. **(P5, 38 YEARS)**<br>Uh huh….we try to explain it to you: if we continually put our fingers in there, you are going to get an infection. By the time you deliver, your baby will come out with a fever. There are instances when we are able to convince, but there are others what will continually worry you throughout. Yet still, we do what we are capable of doing for them (chuckling), and sometimes if you do not attend to the client, it may be true **(P6, 38 YEARS).** |
| Encouraging male involvement | 4 | 5 | Here, most of them are even willing to come around. So, once the person is here, you give the partner a chair and he will be sitting with her and having a conversation. This can even take her mind away from the pain a little and then you teach the partner how the massages are done **(P10, 35 YEARS).**<br>Over here, we try as much as possible to involve the males throughout the process. As such, we normally encourage them to come in and support the women. You know; women can be more cooperative when it is their husband who is giving them sacral massage or when it is their partner who is encouraging them to push the baby out. So, we encourage room-in and involve the men the birthing process **(P7, 32 YEARS).** |

| | | | |
|---|---|---|---|
| | | | he husband (can be made to come in)? Yeah, it is possible. That one, it is possible, because the person will just be in the room. So, during delivery, if the husband is around…. we've allowed one husband to be **(P8, 28 YEARS).**<br>Here because patients are been screened, we do allow their spouses in even if they want to cut the cord, we guide him in doing that **(P9, 51 YEARS)** |
| Sacral massages and reassurance | 7 | 10 | Usually, women in labour are tensed and anxious. As such, it is critical to de-stress them and make them feel comfortable. So, what we do is that, we give them sacral massages. In addition to that, we give them words of encourage so that they (woman) will feel reassured. **(P10, 35 YEARS).**<br>at times we seek the support from the husbands to give them [their wives] the sacral massage, encourage and reassure them **(P3, 35 YEARS).**<br>Some of the patients are emotional. They are often fearful and anxious. Possibly, it is because they have not given birth before but may have heard that the process is painful. For some other clients, the way people may have described the process to them would have put a frightening aura around the birth process for the. In such instances, we try to calm the woman by encouraging and assuring her that she is in safe hand, and that everything is going to be alright. We keep her informed by describing the stages of labour to her. This helps us to calm the person and gain their cooperation because they are now aware of all the stages of birth and all other expectations **(P4, 34 YEARS).**<br>Yeah. I will not tell you exactly how the pain is and progresses, but I will let you know that when it comes to the bearing down, the pain is excruciating. So do these things for the baby. Once you get your baby, you will be fine. So intermittently, I will come and rub your back. I will be giving you sacral massages and I will be teaching you the breathing exercises. Little by little…. whatever you want to do, I will help you to do it the right way. You may want to lie on the floor, but I can help you up and let you sit down **(P7, 32 YEARS).** |
| *Actions that promote dignified care* | | | |

| | | | |
|---|---|---|---|
| Ensuring confidentiality | 3 | 4 | Oh, in such situations, I will be able to admit you and put you in a bed and then when I am taking the FH, your vitals and such inside, I can ask you that. It doesn't exactly have to be at the time of admission that I have to get all the required information, for when I am taking the vitals, the relative is not with us. Uh huh, so I am alone with you in the cubicle, 'OK, Sister, please, for the medications, have you started treatment? Have you done this or that?' and when we get to such a stage, the client is capable of telling you everything, knowing it is just between the two of you **(P4, 34 YEARS).**<br>If you can't provide privacy, you have to talk in a lower tone because if it is abortion and you alarm it for relatives to hear they can divorce her. All you need is to talk under tone to prevent others from hearing **(P9, 51 YEARS).** |
| Provide information and seek consent | 4 | 5 | Well, 'Please Madam, I am here do something for you. I am here to listen to the baby's heartbeat, how well it is beating', or if it is for a VE, 'I am about to put my fingers down there to see how opened your cervix is. That will help us determine the best way to help you in this'. So, you will explain these to her. If she gives you the go-ahead, then you go about it **(P4, 34 YEARS).**<br>If it is MagSulf (magnezium sulphate) – MagSulf is given every four (4) hours – you tell the patient that…. they, the patients refer to MagSulf as 'the needle that extremely burns', so 'Madam, I am about to give you the injection that is extremely painful or burns. You seek for their consent before you do that: everything you do for a patient; I think you have to explain and seek consent **(P8, 28 years).**<br>Like…. Mmmmm……I see it that anything you have to do, you have to make the client aware, you have to tell her and explain what you are about to do and why you are to do it, if she will give you the permission **(P10, 35 years).** |
| Refer mothers who cannot pay bills to social welfare | 6 | 7 | Maybe when the client got here, she is saying she cannot pay. And the time is due for us to discharge her for her to go home, and then the accountant will in and he has to involve the social welfare, and then they come in to find a |

| | | | |
|---|---|---|---|
| | | | way to get the money, or if they have to devise a gradual payment method, then they do that **(P10, 35 YEARS).**<br>if she doesn't pay the bill, she is not allowed to go. So, if you give the case to the social welfare, then I think you've done your part **(P4, 34 YEARS).**<br>If a client cannot pay her bills, we turn her over to the social welfare department. Yeah. So, we no longer detain them as we use to……. **(P5, 38 years).**<br>We come in when the patient tells us that maybe 'I was charged a thousand cedis (1000GHC), I have five hundred (500GHC). So our new approach as midwives is to involve the social welfare department ……'. So, we refer them to social welfare. Then they take it over from there. But before the RMC training, we use to detain them……yes…. of course, because that is the hospital protocol **(P8, 28 years).**<br>Yes, they all pay. Is the general side that I saw one but we called the social welfare but later someone came to visit a client and paid for the bills when they heard her case **(P9, 51 YEARS).** |
| *Actions that promote respectful communication* | | | |
| Ask relatives to excuse patient-provider conversations | 5 | 6 | What we can do is to make the relative excuse us or step aside **(P3, 35 YEARS).**<br>When referrals come at the same time. Even if is second stage we attend to them first before we take other history, at times we ask other patient to excuse us if only the energy is there to wake and excuse us **(P2, 37 years).**<br>So, you just have to excuse the relatives…allow the relatives to excuse you so that the woman can be, the person can be truthful enough to tell you whatever (there is). Because, some people wouldn't feel comfortable if their husbands or their spouses are around, or their family members, their relatives are around to tell you whatever, especially when they have their top-sending **(P8, 28 years).**<br>But if she sees a relative around…I walked up and told the relatives to excuse us for a while so we can get closer to the client and take better care of her, but they got offended even though I did not say it in a bad way **(P4, 34 years).** |

| | | | |
|---|---|---|---|
| *Introducing ourselves as caregivers* | 2 | 2 | For communication we're very good at it. After shift handing-over, we introduce ourselves to them so if they've problems they complain to us if we need to call a doctor we do if we can handle it, we do the needful **(P3, 35 years).**<br>Yeah. So, when we introduce ourselves to the clients, even if not by name, then when you go to perform a procedure for the client, she already knows you are part of the staffs and so she already has confidence in you. Then she goes like 'Madam, what is your name?' and you tell her your name and maybe she also says 'I am also called this'. Ahaaa….and then you proceed to conduct the care you have to give to her **(P4, 34 years).** |
| *Challenges to providing emotional support* | | | |
| Inadequate staffing | 9 | 13 | …it got to do with the inadequate staff. If we're not busy we can attend to the patient promptly but if we are two (2) on duty and we are all busy we can't attend to the third person. We attend to the emergency cases and later to those that can hold on with their condition but if there were to be enough staff, we can attend to all **(P1, 34 years).**<br>…with respect to emotional support, it is quite challenging because of our staff strength. There could be more labour cases which makes it difficult to promptly attend to a patient's emotional support one after the other but we reassure them **(P3, 35 years).**<br>…sometimes, here [referring to the hospital], we do seventeen (17) deliveries at night so in case there's workload and you call we can't attend to you; [this is] not intentional though, but the midwives [are] not enough **(P11, 26 years).**<br>For example, if the number of nurse-to-patient ratio, if the number of patients is too high, like one nurse caring for maybe three (3) or two (2) labour cases at the same time and there are other patients at the ward that you are taking care of, you just can't concentrate on that particular (labour) patient. So, you will leave her a little and go and take care of the other patients. So, at times, they will mention your name, and at times they will 'please help me, I need you', but maybe you are going to share |

| | | | |
|---|---|---|---|
| | | | medication at that time. So, you won't be around to meet that need **(P10, 35 YEARS).** Well, my challenges….one is, here (KATH) is a referral hospital, so they do refer cases more, so if the…. and we mostly come for night duties. Especially (at) night, we are four midwives. If the clients are more than…. sometimes they can be ten (10) or more, so you can't attend to all. So, if the clients are a lot, mostly, it is very difficult for us to be able to take care of every one of them **(P6, 38 YEARS).** |
| Non-cooperation from patients | 9 | 13 | Some patients don't cooperate even difficult to check foetal heart (FH) just because here is an official ward you can't really do much but wait patiently if they're ready you intervene **(P1, 34 YEARS).** Some patients don't comply with instructions. How do we apply the massage? We do the sacral massage but some mothers don't cooperate so we can't do it **(P11, 26 years).** Let's say there are some patients, you approach them in this way: 'Ma'am, the pain you are going through…. especially after labour has started…. that is what propels the baby down the birth channel to the outside, but no matter what you say to such a patient, she will ignore you. No matter what you say, she will ignore you. It is rather what your advice against that she will actually do…. And it seems like whatever you've been saying to her does not really register in her mind but rather passes over her ears. So, it can cause you to be reluctant: you can actually make up your mind not say anything further **(P5, 38 years).** What I can say is, it is also their relatives. I don't know, because this facility is a referral centre when they are being referred and then they come here, even when it is not labour, I mean it is a condition they came in with, the relatives don't give us that space. You tell them to, maybe the patient is in pain, they are around, you tell them (relatives) to go, they (patients) are in your hands now, you are taking care of them, together with the doctors. Every point in time, you see a relative trooping in, especially when they see that the patient that they brought, the relative that they brought, the person is in pain, they (relatives) want to be involved in whatever is going on. I |

| | | | |
|---|---|---|---|
| | | | am not saying that they shouldn't be involved, but I think so far as you've brought the patient into the facility, you've left her in the care of the doctor and the midwife or the nurse. So, whatever is going on, they should just allow us to do our job. Yeah, they should help us do it as they should just give us that space, that room to perform our jobs and after that, we will communicate whatever is going on to them **(P8, 28 years).** |
| Work load | 7 | 7 | To me my major challenge is when I have more than one labour case. I might perform sacral massage on a patient but can't do same for the other at the same time **(P1, 34 YEARS).**<br>Oh, sometimes here we do seventeen (17) deliveries at night so in case there's workload and you call we can't attend to you not intentional though but the midwives not enough **(P11, 26 YEARS).**<br>the challenges maybe will entail work overload; if the amount to be done is a little over our limit, and if I have already attended to you, I have observed everything to be in order, I have done what needs to be done and you continually call me while someone else needs my help, I have to go and take care of the other patients too. You, see? So, it is imperative that you the one person, you as the midwife, you have to apportion your time well so it is possible to look after every client and everyone's care is covered **(P4, 34 YEARS).**<br>But because of the workload, the nurse may forget about it. The patient will come again and come and ask but the tonation with which the nurse may respond 'I said I will do it for you', and the patient may feel so bad (laughing)….'I said I will do it for you!' **(P8, 28 YEARS).** |
| Inadequate material resources and limited space | 8 | 10 | So far, the hospital has no waiting room for relatives so if they come, they've to wait outside **(P1, 34 years).**<br>We have two (2) delivery beds in one room and two (2) women delivering at the same time with no curtains. Now here we have curtains. But the entrance is the problem where the husband will pass because the labour ward is connected to the theatre so entry is a problem **(P2, 37 years).** |

| | | | |
|---|---|---|---|
| | | | So, if two (2) or three (3) ladies become 'full' simultaneously, we will do the others on beds, and the others right here (indicating). Therefore, for the ones that we have to perform our duties on a bed, the husband cannot practically be there, because, right beside the wife, there will be another patient lying there. If you do that, you will be invading someone else's privacy **(P5, 38 years).** |
| Partner's lack of courage | 2 | 2 | Many men don't like it that way they don't want to see their wife going through that pain. I remember I invited a husband and he even collapsed before seeing the baby's head (laughs). Some will refuse if you offer the opportunity because he can't watch. Few ones that are eager we have a way of letting them in. I gave some husbands the opportunity **(P2, 37 years).**<br>There are some fathers who, when they see the baby's head popping up and we are about to give an episiotomy, they vanish. Most are not able to endure that, but for those who are willing and ready to support their wives, they will hold the hand and stay put while we perform our duties **(P7, 32 YEARS).** |
| *Challenges to offering respectful communication* | | | |
| Attitudes of midwives | 6 | 6 | At times (laughs) let me say is their character or some encounter she had before come to work so it annoys her seeing you the patient. The moment you ask question she will scold you not really good but not all of us are like that so we have to advise ourselves **(P12, 30 YEARS).**<br>Apart from the skills we acquired we should always add attitude to it. Some lack attitude to practice as a pragmatic midwife. Personal introduction doesn't come in from the beginning of midwifery. We always concentrate on baby to ensure safe delivery as mom is in to deliver you can't keep long with all introduction. In my view it is time wasting though it is supposed to be like that but our mentality. It doesn't take much time to introduce yourself but not common **(P2, 37 YEARS).**<br>Yes. Some of us, especially, some of us don't know how to talk. Let me generalize that: some of us do not know how to talk. But I think it also boils down to you the nurse, because you are the care provider as at the |

| | | | |
|---|---|---|---|
| | | | moment…. taking care of the patient. So, you being a challenge to the patient and the patient also being a challenge (laughing while saying this) to you, at the end of the day, both of you cannot get to the desired destination that you want to get to **(P8, 28 YEARS).**<br>Some midwives call patients by condition they have. Professionally it is not done. A patient has a real name and must be called by that not her condition. We have to put a stop to that it is a condition and won't stay forever **(P9, 51 YEARS).** |
| Language barrier | 2 | 2 | In some cases, it is the language; there are situations in which the language being spoken by the client is not understood by me. She speaks a different language: she doesn't speak Twi and neither does she speak English. In a scenario like that, it has to fall on the family member present who speaks the language (of the health worker). So, you speak to the relative, and the relative then translates for the client **(P10, 35 YEARS).**<br>Erm...it depends on the level of communication of the relatives maybe language barrier you've to explain things to their understanding but at times you've to seek the patients consent rather so that she can relay the information to relatives to her satisfaction **(P2, 37 YEARS).**<br>I quite remember there was one patient that was to be transfused, whatever the doctor said she replied no to it. The doctor said it in Ghanaian language (twi) that, she doesn't have blood so she has to be transfused and she insisted that she won't allow. The doctor wrote that the patient refused to be transfused but later we asked her why and what church she attend because most church don't accept blood. So, we asked her and she said we've already informed her she is suffering from high blood pressure why then should she take in blood again? That's miscommunication because we told her she has high bp and we said it in twi "mogyamuroso" in hospital sense is a rise in pressure but to her it means the blood has increased. So, if the doctor got the time to explain to her, she would have realized the actual meaning if not the |

| | | | |
|---|---|---|---|
| | | | extra communication we had we wouldn't have known **(P1, 34 YEARS).** |
| Forgetfulness | 1 | 1 | Possibly it maybe forgetfulness if not we do but is thirty percent (30%) or we're just skipping it. If not there's no challenges because if there's an emergency one will attend to it and the rest will be attending to normal cases **(P1, 34 YEARS).** |
| Patriarchal cultural beliefs | 1 | 2 | Some patient prefers seeking consent from husband before we begin any procedure so if the husband is not around, we have to wait for him to be around before starting any Procedure **(P1, 34 YEARS).** |
| *Challenges to respectful and dignified care* | | | |
| Logistical constraint on alternative birthing positions | 11 | 14 | We don't have the necessary equipment and the way clients may behave, if we decide to improvise on the squatting, it is not going to go well. It will not work well at all **(P7, 32 years).**<br>There is position like the squatting position but here we don't have the resources to support the squat birthing. We are used to the lithotomy and all the delivery beds are in that form. We would have adapted to patient's preferred position such as squatting if we had the needed equipment **(P2, 37 years).**<br>For water birth, hmmmm…… we are aware of such position. However, we don't have it [equipment] here. Nothing even shows that we are actually preparing ourselves to do water birthing! Should a patient request for this, then, hmmm…… we cannot meet this need. Let us see what the future holds for all other birthing positions. **(P1, 34 years).**<br>So most of our positions have turned to…..(lithotomy?), so due to the lack of beds, we are not able to provide the position a client may prefer. A client may tell you she wants the squatting position, and if we had the beds and those other things, if they were available, it would have helped. But we don't have those. It seems all our beds are those in which the client will lie down and put her feet up – the same lithotomy position – and as a result, this is a challenge **(P10, 35 YEARS).**<br>When we came to the workshop, we talked about various positions, but we have only one here. So, if the client |

| | | | |
|---|---|---|---|
| | | | maybe wants squatting, or that position with the stool you talked about, it becomes something else for you. For you the midwife: the fact that you don't have the equipment means you can't offer that service to the client and so she has to resort to the same lithotomy position **(P4, 34 YEARS).**<br>Uh huh……but because of the lack of resources, we continue to adapt to the old methods we are used to. Thus, mostly, we are limited to the two (2) methods: either the client laying on her back and we encouraging her to push or, sometimes they will say 'Madam, I did all my deliveries at home, and I squat'. No matter what you do to her, she is squatting! (Laughing). So, you just leave her alone, and you place stuff on the ground…so that the baby doesn't get hurt. So, if we get the equipment…. the accessories and other things, then we can practice the alternatives **(P6, 38 YEARS).** |
| Facility environment limits privacy and movements | 6 | 8 | ……ahaaa, so they are all bunched together inside and if you are working on a patient and you even use the screens, there are holes within the screens so someone can see through these (holes) and take a peep at naked patients. This too doesn't help us **(P5, 38 years).**<br>the environment is not spacious enough to accommodate fee movement. There is always overcrowding due to the patient turn out. Hence, so we restrict walking around **(P9, 51 years).**<br>Ideally, the hospital has to provide drapes but we don't have so we cover clients with their own sheet. Assuming the client comes in an emergency with no clothes, then ensuring privacy becomes a challenge **(P3, 35 years).**<br>As for the drapes, we don't have any in this ward. So, the alternative we have here is the screen. So, we screen the patient and we do our thing **(P5, 38 years).**<br>The drape itself isn't available (smiles) and let say the ward has only three (3) and it has been used. With my previous ward, the screens were scarce and even if it is available, it is faulty. Thus, providing privacy becomes a challenge **(P1, 34 years).** |
| Condition of clients limits movements | 4 | 4 | Yes, when the fluid comes out so you can't allow her to move about with the fear that as the fluid leaks it could |

| | | | |
|---|---|---|---|
| | | | rupture for the cord to slip to cause cord prolapse that's why we restrict them but if everything is fine without rupture, they're allowed to move around because the force of gravity is even necessary so we encourage them to walk **(P3, 35 years).**<br>Well, when it comes to walking, you are allowed to walk but it depends on each stage and what you expect from the client. Let's say the client is at eight cm (8cm) …8-9, at that stage, this is difficult. It is a transitional stage and she is traumatized and such, there is also the possibility of prolapse, and other complications too **(P4, 34 years).**<br>The challenge is when the baby's head is not well attached to the cervix and has rupture membranes for that if care is not taken her cord can slip to affect the baby. Apart from that if everything is intact, she can move freely and also when she is seven centimetres (7cm) dilated she can unaware push the baby down if not she can move freely **(P9, 51 YEARS).** |
| *Recommendations for promoting emotional support* | | | |
| Increase staff strength | 11 | 11 | More staff needs to be employed. We cannot have one (1) the patient is to one (1) midwife but at least there should be extra so that activities can be shared among us **(P1, 34 years).**<br>We need to increase staff and some staff needs to change their attitude. We are not the same. Some will work hard others will not so mostly the patient expect the hard workers to treat them but you may be tired. We have to advise ourselves not all of us but at times we are the problem **(P12, 30 years).**<br>Staff: if we are many, it will help a lot…. because there is a lot of work to be done here. If we had the right number, it will at least help us to do the work commensurate to what is expected of us and we can give all the (emotional) support that we should give **(P5, 38 YEARS).**<br>That we can be made a little more? Yeah, that the number of staff be increased a little more so we can, then educate us more. There some that if you don't talk to them incessantly…there are some nurses that if you do not continually educate, if they already have a certain innate character, they cannot let it go. But they more you give |

| | | | |
|---|---|---|---|
| | | | them instances like ‘someone did this, and this was what resulted to her’, for I am personally fearful of that particular scenario, a patient putting a curse on me **(P7, 32 YEARS).** |
| Support and intensify maternal education | 6 | 11 | I think mothers should be psyched during antenatal care (ANC). From my experience, I delivered not long ago and labour is not easy my mom told me that labour pain is unbearable so I psyched my mind so mothers need to be psyched and also check their appearance and it will help **(P11, 26 YEARS).**<br>Well, concerning the emotional….in our ward, what I will talk of is flip charts. Yeah. You show the pictures to the client and explain along, ‘right here, you can see she is labouring to breath, right here, you can see this and that is what is happening to her’, you see? So, this can encourage the client to understand she is OK. This is what is happening to everyone so it is not as if mine is abnormal or something of the sort **(P4, 34 YEARS).**<br>Hmmmmm…. we have to really intensify education. The last time, we were discussing it here that we needed education for the clients like on radios and such. Because, I do not know why…it seems they allow superstition …. I have observed that they allow their beliefs outside and their influences to be more important to them. People influence them negatively, especially and such **(P6, 38 YEARS).** |
| *Recommendations for promoting respectful communication* | | | |
| Peer monitoring | 2 | 2 | Hmm… for now I think we have to be each other’s keeper. There may be somethings that you will do and it will feel odd. But if we check on each other and become each other’s keeper, then we will be able to help ourselves at the ward. We can talk to ourselves, and also involve the senior midwives so that each student nurses and rotational nurses will be practicing professionalism in communicating to patients when they come here to give birth. All these are taught in school and doesn’t need any workshop or training. If you treat a patient right, they deem it much offer you’ve done them. **(P2, 37 years).** |

| | | | |
|---|---|---|---|
| | | | No but with respectful maternal care we've to be each other's keeper so patients should take it easy with us in order for us to deliver care to them **(P3, 35 years)** |
| Demonstrating good attitudes | 1 | 1 | We don't need anything special but it is up to us to have good attitude and know how to deal with patients. We have to educate them on when to ask questions it may seem emergency to them but they've to bear with us. Most of the problem is our attitude so no matter what we've to portray good attitude towards our patient **(P3, 35 years).** |
| *Recommendations for promoting respectful and dignified care* | | | |
| Logistical support for alternative birthing | 9 | 10 | The hospital has to acquire all the necessary equipment that will enable us to practice alternative birthing like the water birth **(P1, 34 years).**<br>We need the beds and the instruments they will use for those positions. As long as we have those, we will allow them to use them **(P10, 35 years).**<br>Oh OK, we have to get the different equipment performing those positions, like the birthing chair you talked about when we came (for the workshop) **(P4, 34 years).**<br>Please, I believe the bed itself: I learnt for the squatting position, there is a bed specifically designed for that. And the water birth too, I believe there is a small balloon-like device that we can use for that. So, if we get such devices here, we can ask the women to choose the position they may desire to give birth in **(P5, 38 YEARS).**<br>Mmmmm…when it comes to resources, we need a lot. Let's say we want to do the water birth or the other positions, unless we get the requisite equipment or resources to go with the positions **(P6, 38 YEARS).** |
| Capacity building and trainings | 5 | 5 | Then, there should be a training on that [alternative birthing positions] for midwives. Though we've been taught about the new ones in school but we never practiced it so if the hospital is to provide it has to organise workshop for us **(P1, 34 years).**<br>when I had not yet come (to the workshop) to learn about respectful care, some of my attitudes and behaviours were not really optimal but once I came and got educated on these, things have changed. And a lot of things have changed. Therefore, we should go to the district, and other |

| | | | |
|---|---|---|---|
| | | | sectors and educate them the more, and even if it is possible, let us take the education to the schools so that by the time the person is coming (the healthcare worker is being sent to the ward), she would have picked it (the desirable concepts of respectful maternal care) already. And we need to continually re-educate, so our minds would be drawn to certain things all the time **(P10, 35 years).**<br>We need to get a lot more training, because in school, it was the lithotomy that we were trained much on about the delivery. Aha, so when you talk about squatting, on all fours, and such delivery positions, we've learnt it but in conducting delivery in such positions, it is not all of us who have ever had experiences with these. Ahaa. …but for lithotomy, it seems it is a procedure you've been sat down, taught and emphasized on, so you are conversant with it and you've done a lot of deliveries on such positions before. So, I believe if we can be given a lot of training on these in the workshops in such forms of delivery, and practicals are added **(P4, 34 YEARS).** |
| Logistical support for privacy and free movement | 3 | 6 | I think we need individual room with a second stage bed so that from first stage she will be there till delivery so that whoever is around to support would be allowed till she delivers. But with many in a room we can't allow for someone to see one's nakedness (laughs) **(P2, 37 YEARS).**<br>Yeah, when the screens are not enough, we can use the curtains. All you have to do is draw the curtains. So, if we were provided with more curtains or if they gave us more screens, we could ensure privacy. But albeit the problems, we do ensure privacy all the same **(P6, 38 years).**<br>Maybe in our ward, we should separate the labour cases from our ward to a different place, or let's say for Cubicle One (1), let's make it into a cubicle for only labour cases. So, we can use curtains to separate the place into individual rooms for each client that comes in. So, if you are in labour, your relative can stand beside you during the process **(P5, 38 years).** |
| Motivating midwives | 2 | 2 | Mm we have talked about all but I will add up that the hospital should add a bit of motivation to staffs although |

| | | | |
|---|---|---|---|
| | | | they are being paid for all they do but a bit of motivation will do to encourage us because at times you will realize they don't appreciate what we are doing **(P1, 34 years).** Also. staff motivation is a factor that people don't put much effort to render services to patient. I'm not saying is a right way but if I'm sick as a midwife and comes to my facility and they have to take money before treating me if I don't have money, they won't treat me but I work here. If I'm sick and I will pay my bills that the hospital doesn't have any policy to cater for me the health personnel then I will get someone to give me excuse duty for two (2) weeks and rest do you understand please? If I stay home staff strength will decrease but if hospital cater you can give days to rest even surgery without insurance and some patients who are not health workers are covered with their job insurance. If your husband's insurance doesn't cover you, you'll work with bitterness which is not good. You come to work unbitted the patient issue doesn't concern you. If you calculate three hundred Ghana cedis (300gh) for transportation and an unforeseen thing happens you have to pay. It really disturbing and sad **(P2, 37 YEARS).** |
