# Supplementary material for: Promoting respectful maternity care: challenges and prospects from the perspectives of midwives at a tertiary health facility in Ghana
Source: BMC Pregnancy Childbirth. 2022 May 31;22:451. doi: 10.1186/s12884-022-04786-w (PMC9153163; doi:10.1186/s12884-022-04786-w)
Supplement: Supplementary file 2 — Additional file 2. [file 12884_2022_4786_MOESM2_ESM.pdf]

Respectful Maternity Care Interview Protocol Midwives

Interviewer introduction note: My name is………………………………………

Demographic details:

1. Age:
2. Education qualification:
3. Years of experience:
4. Parity status:
5. Married: A: Never married

   B: Currently married

   C: Previously Married

	Challenges you encountered in the past in performing this duty/challenges you foresee encountering (what is likely to prevent you from performing this duty). Challenges can be under the following themes: challenge due to clients' conditions (attitudes, age, neatness of appearance, etc.), institutional physical and policy environment, and provider conditions (attitudes, beliefs, values, etc)	*What provisions or assistance do you think, when made available, will empower you to discharge this duty effectively.*	*What are your reservations on this duty? State specific suggestions or alternatives. [If none, ask interviewee to give a general remark on the duty.]*
**Emotional support**			
Childbearing women may have many thoughts, including those of fears and anxiety. So, you should reassure them of how safe the childbirth process is. Massage			

the woman in a comforting manner and talk to her to alleviate fears.<br>[probes: we should ask real world examples here to probe]			
Allow birth companions during labour and delivery/postpartum.<br>[probes: what of the VIP ward—cost etc]			
Timely response/action to clients' needs when required for support in<br>labour or postpartum.<br>[probes: we should ask real world examples here to probe]			
**Respectful communication**			
Introduce yourself as a caregiver, ask of the woman's name and address her by name, ask about her needs or questions, and attentively listen to the concern of the woman.<br>[probes: we should ask real world examples here to probe]			
Explain ward operations to clients and their relatives in a respectful tone.<br>[probes: we should ask real world examples here to probe]			
Explain care procedures clearly and obtain consent from the woman/family before performing any.<br>[probes: we should ask real world examples here to probe]			
Taking woman's medical records without the presence of family members and other childbearing women. [probes: confidentiality—we should ask real world examples here to probe]			

Seek and obtain consent from woman before disclosing her medical records to family members.<br>[probes: we should ask real world examples here to probe]			
**Respectful and dignified care**			
Draping woman before and after vaginal examination.<br>[probes: we should ask real world examples here to probe]			
Not pinching in-between the thighs or beating or slapping the woman regardless of the provocation from her to get her to do what you want her to do.<br>[probes: we should ask real world examples here to probe]			
Not tying (no use of physical restrains) women with straps on the delivery bed before performing examinations.<br>[probes: we should ask real world examples here to probe]			
Guiding the woman to deliver in the position of her choosing.<br>[probes: we should ask real world examples here to probe]			
Assist and allow the woman to move freely in the labour ward.<br>[probes: we should ask real world examples here to probe]			
Using curtain/screens to separate woman from others before uncovering them to perform examinations/giving injections.<br>[probes: we should ask real world examples here to probe]			

Not detaining the woman in facility due to lack of payment of facility fees or any other reason.<br>[probes: we should ask real world examples here to probe]			
